# Indigenous Genomic Databases: Pragmatic Considerations and Cultural Contexts

**DOI:** 10.3389/fpubh.2020.00111

**Published:** 2020-04-24

**Authors:** Nadine Rena Caron, Meck Chongo, Maui Hudson, Laura Arbour, Wyeth W. Wasserman, Stephen Robertson, Solenne Correard, Phillip Wilcox

**Affiliations:** ^1^Department of Medical Genetics, University of British Columbia, Vancouver, BC, Canada; ^2^Genome Sciences Center, British Columbia Cancer Agency, Vancouver, BC, Canada; ^3^Northern Medical Program, University of Northern British Columbia Canada, Prince George, BC, Canada; ^4^Faculty of Māori and Indigenous Studies, University of Waikato, Hamilton, New Zealand; ^5^Department of Mathematics & Statistics, University of Otago, Dunedin, New Zealand

**Keywords:** indigenous, genomics, variant, databases, maori, variome

## Abstract

The potential to grow genomic knowledge and harness the subsequent clinical benefits has escalated the building of background variant databases (BVDs) for genetic diagnosis across the globe. Alongside the upsurge of this precision medicine, potential benefits have been highlighted for both rare genetic conditions and other diagnoses. However, with the ever-present “genomic divide,” Indigenous peoples globally have valid concerns as they endure comparatively greater health disparities but stand to benefit the least from these novel scientific discoveries and progress in healthcare. The paucity of Indigenous healthcare providers and researchers in these fields contributes to this genomic divide both in access to, and availability of culturally safe, relevant and respectful healthcare using this genetic knowledge. The vital quest to provide equitable clinical research, and provision and use of genomic services and technologies provides a strong rationale for building BVDs for Indigenous peoples. Such tools would ground their representation and participation in accompanying genomic health research and benefit acquisition. We describe two, independent but highly similar initiatives–the “Silent Genomes” in Canada and the “Aotearoa Variome” in New Zealand–as exemplars that have had to address the aforementioned issues and work to create Indigenous BVDs with these populations. Taking into account the baseline inequities in genomic medicine for Indigenous populations and the ongoing challenges of implementing genomic research with Indigenous communities, we provide a rationale for multiple changes required that will assure communities represented in BVDs, as well as Indigenous researchers, that their participation will maximize benefits and minimize risk.

## Indigenous Genomic Databases: Pragmatic Considerations and Cultural Contexts

The push for precision medicine is escalating in countries with resources to engage in genomic and genetic research in health-related fields (1–3). Reported success stories highlight potential benefits for both rare genetic conditions and other diagnoses such as cancer. Combining this growing knowledge with genomic background variant databases (BVDs) has the ultimate potential of improving health care along the spectrum of risk stratification, diagnostic tools, treatment options, and clinical outcomes. With such potential benefits, it is imperative that this progress is continually checked and ultimately bridged through equitable economic investments, clinical research, and provision and use of genomic services and technologies globally (4, 5). The danger is that the predicted “genomic divide”^1^ will grow, as those who endure the greatest health disparities will benefit the least from these scientific discoveries and progress in healthcare. Example populations with such valid concerns include Indigenous peoples globally. This paper examines these perspectives in two high income countries: Canada and New Zealand.

There are successful examples of genetic and/or genomic research with Indigenous populations. In British Columbia (BC), Canada, cooperative efforts in research exploring the elevated prevalence of sudden cardiac death in members of the Gitxsan First Nation led to identification of a genetic variant that confers an increased susceptibility to arrhythmia and sudden death, the Long Q-T syndrome (6). Following this discovery, effective diagnosis and medical care for the affected Gitxsan community members could then be possible alongside further research to understand the complexities of the condition (7, 8). In New Zealand, research on gout has been conducted in consultation and collaboration with the Māori tribe, Ngāti Porou, via its health provider, the Ngāti Porou Hauora Charitable Trust (9). This led to identification of genetic variants associated with high levels of serum urate and gout (10, 11), and verification that gout has a heritable basis. This knowledge has not only led to improved diagnosis and treatment, but more importantly for tribal members, has de-stigmatized gout as a disease arising from heritable genetic factors rather than a result of poor lifestyle choices (12, 13). This also led to increased adherence to allopurinol dosing—which reduces serum urate and therefore gout—thus permitting safe consumption of traditional Māori foods that had previously been identified as triggers of gout attacks (14).

Unfortunately, success stories co-exist with negative, exploitative, stigmatizing, disempowering, and/or culturally insensitive examples (15, 16). One example of such genomic research is that done with the Nuu-chah-nulth peoples in BC, Canada, whose blood samples were originally collected with the understanding that research would be done to elucidate the reason for their high incidence and severity of rheumatoid arthritis. Instead, DNA was used to study human migration and retroviruses (17). Another example is the controversial “warrior” gene research conducted on Māori in New Zealand (18), whose researchers claimed—based on a small sample size—that supposedly higher frequencies of a monoamine oxidase gene variant previously shown to be associated with aggressive behavior in non-Indigenous populations, explained violent behaviors exhibited by some Māori. This research was widely condemned (19–22) for reinforcing inappropriately negative stereotypes of Māori as being aggressive in nature.

It is a grave concern that Indigenous peoples currently have neither equitable access to medical geneticists nor genomic and genetic research that optimizes these health care resources (23, 24). If research does indeed carry the potential beneficial impact we aim for, then surely demand for equitable distribution of research funding, infrastructure for sustainability, and the necessary development of capacity for Indigenous peoples to participate makes sense. Moreover, advocates for genomic medicine need to learn from these histories of genetics/genomics within Indigenous populations and consider what is needed to deliver the benefits and opportunities of genomic science. This can be done by incorporating what is necessary to both create the affiliated genomic resources and utilize them in a culturally safe, relevant, and respectful way. The model of research needs to change to fit what Indigenous peoples, our ancestors and our future generations deserve (25).

## Reasons for Health Inequities in Genomic Medicine in Indigenous Populations

Genomic medicine is poised to generate escalating health benefits. Genome sequencing can yield clinically-relevant data with direct applicability in real-time patient care with an impressive “bench-to-bedside” pathway. Healthcare inequities hinder efforts tackling health issues specific to Indigenous populations. The challenge is to ensure that the option to engage in genomic research and clinical care is equitably distributed. The ability of Indigenous peoples to have the same access to genomic tools for diagnosis and the capacity to have choice in this scientific space is therefore crucial.

The Genome Aggregation Database (gnomAD), used mostly as a reference tool to interpret sequencing data and understand variants associated with disease globally, is not generalizable (23, 26). GnomAD lacks representation of Indigenous populations and therefore is less relevant to these populations—and may be misleading or even detrimental (27, 28). Without this representation, the success and benefits of genomic medicine will be disproportionately less. For this reason, efforts are being undertaken around the globe to produce BVDs for previously excluded populations recognizing that both affiliated policies and scientific infrastructure are required (29–32).

Further, there is lack of consideration of Indigenous communities as reflected in paucity of Indigenous scientists, genomic researchers, medical geneticists, genetic counselors, health care practitioners (3, 15, 33, 34), and personnel in research organizations, and journal review/editorial committees. Such capacity is needed to spearhead genomic research and clinical applications for equity to be achieved.

In this discourse, we recognize that Indigenous peoples' health is globally shaped by intergenerational effects of colonization and ongoing inequitable social practices and policies, structural violence, and institutional discrimination and racism (35). The examples of historical violations in genomic research ethics and the persistent lack of policies or governance structures for Indigenous peoples' genomic data reflects the multitude of other negative colonization experiences, and therefore justifies the valid concerns and reluctance by some Indigenous communities to join genomic research projects. Among others, we highlight two initiatives with teams working to find solutions through Indigenous lenses.

## A Tale of Two Initiatives for Indigenous Databases

These two developing initiatives aim to bridge the “genomic divide.” While they are half a world apart and developed independently, there are remarkable similarities in their rationales, purpose and emerging directions for implementing results. The projects are (1) the “Silent Genomes” and the proposed Indigenous BVD in Canada (36), and (2) the “Aotearoa Variome” in New Zealand (37) (see Table 1). Below, we describe these similarities (and some differences), as exemplars of how research genomic databases may be established in the future.

**Table 1:** Silent Genomes and the Aotearoa Variome projects: in evolution.

	**Silent genomes (Canada)**	**Aotearoa variome (New Zealand)**
1) Background	• Began April 1st, 2018, but officially launched July 18th, 2018 in Victoria, BC through a “Gathering Ceremony” (https://www.bcchr.ca/silent-genomes-project/gathering-ceremony) with Canada's Indigenous leaders, peoples, partners, and other stakeholders.	• Multi-party initiative led by University of Otago, began Feb 2018
2) Principal funding agencies	• Genome Canada (GC) • Genome British Columbia • Canadian Institutes of Health Research (CIHR) • Michael Smith Foundation for Health Research (MSFHR) • Provincial Health Services Authority (PHSA) • BC Children's Hospital Foundation • University of British Columbia • Life Labs (scholarship funding) • Illumina (in kind)	• Ministry of Business, Innovation and Employment (MBIE)-funded platform, New Zealand via the Strategic Science Investment Fund via Genomics Aotearoa
3) Funding duration	• 2018–2022	• 2017–2024
Aims	• To work with Indigenous partners to develop best practices for governance over genomic research and clinical care and to address current gaps in Canadian policy in these areas • To enhance equitable access to diagnosis and improve diagnostic success and care. • To catalog background genetic variation in Canada's Indigenous peoples within a novel background variant database (BVD). • Economics of genomic diagnosis in Indigenous populations—cost effectiveness evaluation	• To catalog genetic variants to support disease diagnosis and research into healthcare conditions relevant and important to Māori and Pacific people. • Develop a BVD on all DNA sequence variation within Māori • Accelerate precision medicine for Māori and Pacific peoples in Aotearoa • Develop and implement Māori control over database use
4) Use of BVD	• Clinical application for improved precision of diagnosis • In-house research by Indigenous peoples not yet determined	• Accelerate clinical diagnoses • Develop Māori and Pacific genomic resources for health research • Longer term: in-house research and clinical applications by Māori health entities e.g., Ngāti Porou Hauora
5) What data will not be used for	• Ancestry testing • As a biobank • Any study unless approved by Governance Group	• Direct-to-consumer ancestry testing • Proof of hapu and/or iwi affiliation(s) • Any other study unless approved by Aotearoa Variome Leadership roopu/Governance group
6) Data ownership and control	• Indigenous led Committee providing advice and oversight of BVD for duration of the project • Longer term—must be ongoing Indigenous governance (membership to be discussed [TBD]) through Governance Group set up when Silent Genomes is phased out	• Interim Kaitiaki Governance Group (membership TBD) to regulate and mandate use of resource • Longer term TBD—must be ongoing Māori governance
7) Net outcomes	• Better diagnostics—earlier and efficient disease detection • More “precision medicine”—effective interventions targeted based on knowledge of genetic make-up • Reinforced public health message for some conditions and increase better uptake into diagnosis and care	

### Research Informed by Indigenous Ethical Frameworks

Most importantly, both initiatives have been informed by previously-developed peer-reviewed Indigenous ethical frameworks; (i) “DNA on Loan” (38) informed the Canadian project, and, (ii) Te Mata Ira guidelines for medical genomics with Māori (39) informed the New Zealand project. These had either been derived from, or previously applied to, genomic studies involving Indigenous communities in their respective countries. These guidelines incorporate “cultural logic” derived from cultural traditions and norms of Indigenous communities (40). They aim to enable researchers to position genomic data and science within culturally appropriate overarching research and oversight frameworks that maximize benefits and minimize risks for participating communities. For the Silent Genomes initiative, the potential Indigenous peoples' DNA samples and data generated will have significant links with cultural practices and should be considered “on loan” to the project team for the sole purpose consented and agreed upon. The Aotearoa Variome project uses a slightly different contextualization—that the DNA is a “*takoha*”—a gift of responsibilities, which obligate whoever uses the data to deliver outcomes that are useful and continue conducting research in a culturally appropriate manner.

### Co-designed and Co-led by Indigenous Researchers

These initiatives include Indigenous practitioners of genomics and/or medicine to provide a contextual background more fit for the intended purpose. These initiatives have been informed by the participating communities with benefits designed to be directly relevant to them. Through these initiatives, Indigenous researchers, and leaders, and non-Indigenous collaborators, are working with communities and individuals to create governance structures to guide how Indigenous peoples can be given the choice to participate and ensure their data are used appropriately. Individual consent with family and/or community approval (when applicable) will be obtained. Subsequent DNA sequencing would then generate data to identify inherent genomic variations. Genomic variants present in Indigenous individuals could be cataloged to facilitate early diagnosis, provision of more accurate genetic counseling, implementation of changes in management or therapeutic intervention for healthcare conditions with a potential genetic basis affecting Indigenous peoples (24).

### Indigenous Governance Over Indigenous Data

It is important that the outcomes of these initiatives are equitable, benefit the community, and address Indigenous data sovereignty concerns regarding ethical use and on-going data security (15). To maximize community confidence and motivate participation, these initiatives are Indigenous-led and governed, with co-development of processes that ensure consenting is done effectively and appropriately, and benefits are consistent with Indigenous expectations.

Aotearoa Variome have a Leadership group consisting of Māori health professionals and researchers who provide project oversight and direction, assist with development of governance policies, and community engagement. Silent Genomes is consulting and collaborating with Indigenous partners, communities and leaders about the potential of creating an Indigenous-specific BVD and implementing and sustaining this clinical tool. This project is developing a governance structure for the Indigenous BVD and policy guidelines establishing best practice models for oversight of biological samples and genomic data alongside its International Indigenous Genomics Advisory Council. Indigenous self-determination and ownership will be respected and promoted as researchers engage communities and have them realize control of their own destiny as worthy recipients of health services (25). Depending on the specific consent provisions for each project and community, samples will be stored in a biobank or repatriated for disposal in a culturally appropriate manner (15).

### Incorporating and Acknowledging Indigenous Knowledge

To ensure extant diversity is represented, and progression toward making the tools equitable, the Aotearoa Variome project uses traditional knowledge of genealogy, including historical “*waka”* (voyaging canoes) affiliations, oral histories and Māori tribal affiliations as indicators/guides, under evaluation by Māori experts in “*whakapapa”* (genealogies). Tribal histories and genealogies are typically maintained by Polynesian cultures via various means, including oral traditions such as song and chants. In the case of Māori, this includes the names of the waka and the tipuna (ancestors) who traveled in them to Aotearoa, as well as the identities of their descendants and the histories associated with those descendants (41). Such information provides a basis for assessing the genetic diversity of participants involved in the Variome, thereby ensuring the subsequent resource adequately represents existing diversity within Māori.

Silent Genomes is also engaging with communities for approval for the use of samples already collected and consented for future research. Eight First Nations communities across Canada are participating in the *Canadian Alliance for Healthy Hearts and Minds* cohort study (42). Each community will decide whether they will participate in the planned BVD with the goal of improving the chances of a diagnosis of a single gene disorder. Further discussions are ongoing with representatives of the BC First Nations Health Authority with the potential that BC-based First Nations may consent to participate. Dialogue is ongoing with Inuit and Métis representatives, the other Indigenous populations of Canada. Silent Genomes incorporates the Indigenous-informed “DNA on loan” concept whose emphasis on sacredness of the biological samples extends to the genomic data derived and remains part of its donors. The samples and or the derived data must be repatriated upon request by participants (38).

### Focus on Benefits

These initiatives have overriding purposes and outcomes which culminate in overall emphasis and action to correct health inequities specific to Indigenous populations. Through the generation, interpretation and discussion of Indigenous specific genomic health data, we anticipate improvements in access to and quality of genomic applications in the medical field. This includes the development of catalogs of genetic variants observed in Indigenous peoples to support future genomic diagnoses. Only with the appropriate capability in both research fields and clinical services can effective genomic healthcare be delivered to Indigenous communities. In the Aotearoa Variome study, participants in previously conducted studies will be approached first, to provide DNA. This will have the immediate benefit of enhancing those other studies via provisioning of full genome sequence.

Capacity building in genomic science for Indigenous communities is being supported through programs like SING (Summer Internship for Indigenous Peoples in Genomics) and translational activities associated with Silent Genomes and the Aotearoa Variome. These initiatives strive to foster genomics health literacy in both participants and health care providers. This will help the latter to undertake multiplex integration of genomic information with clinical practice. Further, both projects aim to expand Indigenous leadership, participation and coordination in health genomics research.

## Ongoing Challenges of Implementing Genomic Research in Indigenous Communities

Beyond these specific initiatives described above, there are a range of additional issues that need to be addressed effectively before Indigenous genomic databases are acceptable to Indigenous communities.

### Recognizing There Is No Single Indigenous Perspective

The need to navigate and collaborate with Indigenous peoples in different communities calls for fundamental recognition of the diversity in the communities' history, culture and traditions. This heterogeneity entails differences in comfort levels between communities and within them; there is no single universal perspective. Individuals and communities may show variable enthusiasm to participate in genomic research and varied views on how their data should be managed. Different cultural contexts may thus require specific implementation strategies.

Furthermore, given that Indigenous populations worldwide have varied experiences, the exemplars given here may not be generalizable. This highlights the need for broader, worldwide perspectives that include proportional representation capturing Indigenous peoples' diversity.

### Building Indigenous Capacity and Capability for Future Leadership in Genomic Research

There is urgent need for training and maintaining current leadership to pass on the knowledge and skills to future generations. It is challenging to create, maintain and sustainably provide remuneration for formal and informal leadership positions, and recognize when to create new leadership roles enabling progress reflecting the urgency required. Moreover, genomic research projects with and for Indigenous peoples may attract less than enthusiastic political will and be afforded less than required priority and thus encounter funding shortfalls along the process (33, 43). Meager resources may complicate Indigenous data governance. Associated with this is the need to improve cultural safety and knowledge of non-Indigenous researchers. In general, genomics education at both undergraduate and graduate level excludes aspects such as working with Indigenous communities and underpinning ethical frameworks. This exclusion needs to be rectified as genomic scientists are likely ill-prepared for working with Indigenous communities.

### Recognizing and Ameliorating the Risks to Indigenous Researchers

Many Indigenous peoples are still healing from intergenerational trauma and are faced with ongoing systemic abuses of their rights. They are also wary of previous exploitative and insensitive research practices being repeated. Getting their ongoing support for genomic research is thus a daunting task that needs time but can be enhanced through transparent consent and governance processes. The risk to Indigenous researchers and providers of health services often leads them to carry the weight of being “the trusted one” by one's kin, and risks them being unfairly held accountable for any negative outcomes (44). Moreover, Indigenous researchers are often placed in invidious positions because of conflicting cultural responsibilities, professional requirements, and the power differentials at play. Here, professional requirements—be those of an employer and/or the broader expectations of the science profession—are expected to take precedence over expectations and practices of Indigenous communities that researchers come from. A culturally safe professional environment would recognize such challenges and provide both assistance and understanding to the researchers—therefore maintaining Indigenous researcher's integrity—and keeping open options for further research partnerships with their communities.

### Motivating “Good” Actors/Actions

The approaches to genomic data generation described here place Indigenous communities at the center of the underpinning research. These approaches contrast with studies such as those that attempt to catalog variation for insights into human evolution for curiosity sake (45, 46). In our view, if Indigenous communities are to participate in genome data generation, funding agencies and journals need to adopt practices that embed Indigenous ethical practices, and de-incentivise self-serving behaviors that create negative experiences.

## Summary

Genomic background variant databases (BVDs) offer considerable potential health benefits and this impact is expanding exponentially as technology and scientific inquiry advances. Yet inequities seem to display similar trends, with the “genomic divide” moving from a cautionary tale to a true entity, as described by the World Health Organization. This could not be truer for Indigenous communities, providing a strong rationale for their representation in BVDs and participating in accompanying genomic health research. However, mistrust arising from extensive negative colonization experiences, institutional racism within the science profession, and egregious experiences of medical genomics have often discouraged meaningful participation. This is true for Indigenous peoples in both New Zealand and Canada, among other countries globally where such research has been conducted.

We provide a rationale for multiple changes required among research practitioners and the broader science profession for Indigenous communities (and underrepresented minorities) to benefit from genomic BVDs.

These include: developing a catalog of genetic variants observed in Indigenous peoples and creating links with diagnostic services to help correct health inequities specific to Indigenous populations; improving genomic health literacy, capacity building for Indigenous peoples, their leadership and researchers working in genome science to address future needs and applications for health; developing new culturally safe guidelines, procedures, and policies for engaging communities and bridging the genomic divide; and protecting the rights and interests, and respecting the views of Indigenous participants pertaining to their DNA samples and data generated.

Such changes will assure communities, as well as Indigenous researchers, that their participation will maximize benefits and minimize risk. We describe two independent initiatives that have taken very similar approaches and had to address similar issues, including implementing most of the aforementioned changes. These initiatives provide potential exemplars of how genomic BVDs can be constructed.

## Author Contributions

NC and PW conceived of the concept for this paper. All authors (NC, MC, MH, LA, WW, SR, SC, and PW) contributed to drafting and editing of the manuscript.

## Conflict of Interest

The authors declare that the research was conducted in the absence of any commercial or financial relationships that could be construed as a potential conflict of interest.

## Footnotes

^1^The “Genomic divide” is the disparity between genomic information available for people with European and Asian backgrounds, and that for Indigenous and other populations in genomic studies (World Health Organisation, 2019).
